# Automated surgical step recognition in transurethral bladder tumor resection using artificial intelligence: transfer learning across surgical modalities

**DOI:** 10.3389/frai.2024.1375482

**Published:** 2024-03-07

**Authors:** Ekamjit S. Deol, Matthew K. Tollefson, Alenka Antolin, Maya Zohar, Omri Bar, Danielle Ben-Ayoun, Lance A. Mynderse, Derek J. Lomas, Ross A. Avant, Adam R. Miller, Daniel S. Elliott, Stephen A. Boorjian, Tamir Wolf, Dotan Asselmann, Abhinav Khanna

**Affiliations:** ^1^Department of Urology, Mayo Clinic, Rochester, MN, United States; ^2^theator.io, Palo Alto, CA, United States

**Keywords:** computer vision, automated surgery, surgical intelligence, surgical step recognition, artificial intelligence, endourology, computer-assisted surgery, urology

## Abstract

**Objective:**

Automated surgical step recognition (SSR) using AI has been a catalyst in the “digitization” of surgery. However, progress has been limited to laparoscopy, with relatively few SSR tools in endoscopic surgery. This study aimed to create a SSR model for transurethral resection of bladder tumors (TURBT), leveraging a novel application of transfer learning to reduce video dataset requirements.

**Materials and methods:**

Retrospective surgical videos of TURBT were manually annotated with the following steps of surgery: primary endoscopic evaluation, resection of bladder tumor, and surface coagulation. Manually annotated videos were then utilized to train a novel AI computer vision algorithm to perform automated video annotation of TURBT surgical video, utilizing a transfer-learning technique to pre-train on laparoscopic procedures. Accuracy of AI SSR was determined by comparison to human annotations as the reference standard.

**Results:**

A total of 300 full-length TURBT videos (median 23.96 min; IQR 14.13–41.31 min) were manually annotated with sequential steps of surgery. One hundred and seventy-nine videos served as a training dataset for algorithm development, 44 for internal validation, and 77 as a separate test cohort for evaluating algorithm accuracy. Overall accuracy of AI video analysis was 89.6%. Model accuracy was highest for the primary endoscopic evaluation step (98.2%) and lowest for the surface coagulation step (82.7%).

**Conclusion:**

We developed a fully automated computer vision algorithm for high-accuracy annotation of TURBT surgical videos. This represents the first application of transfer-learning from laparoscopy-based computer vision models into surgical endoscopy, demonstrating the promise of this approach in adapting to new procedure types.

## 1 Introduction

A fundamental goal for the application of artificial intelligence (AI) in surgery has been automated surgical step recognition (SSR) from intraoperative video footage (Jin et al., 2018). Automated detection of the step of surgery is the foundation for a variety of potential applications that may be offered by “intelligent” context-aware computer-assisted surgery systems, such as intraoperative decision-support, surgical teaching and assessment, monitoring surgical progress, and linking intraoperative events to post-operative outcomes (Mascagni et al., 2022). Although there has been considerable progress in SSR, most published studies have focused on laparoscopic procedures because of their standardized procedural workflow, visual clarity, and availability of large datasets needed for model training (Anteby et al., 2021). However, there is a need to expand SSR technology beyond laparoscopy into diverse surgical modalities, including surgical endoscopy.

Urothelial carcinoma of the bladder is the 6th most common solid-organ malignancy in the US (Saginala et al., 2020). Consequently, transurethral resection of bladder tumor (TURBT) is a very commonly performed endoscopic surgery that is integral to bladder cancer diagnosis and management. TURBT is an ideal prototype for the study of SSR due to its structured sequence of definable steps that are largely standardized across surgeons and across tumor types. Similar to modern laparoscopic procedures, endourologic procedures generate high-quality videos that can be annotated for analysis. Moreover, SSR in TURBT has immediate demonstrable value in downstream applications such as operating room scheduling (Guédon et al., 2021), post-operative reporting (Khanna et al., 2023), and billing (Flynn and Allen, 2004). However, the unique challenges posed by endourologic procedures, including the complexity of urinary tract anatomy, potential camera view occlusion by blood and debris, variations in fluid medium textures, and patient-specific factors all necessitate the development of specialized algorithms and techniques for SSR in the endoscopic setting. Overcoming these challenges may serve as a valuable proof-of-concept for expanding the scope of SSR technology beyond just laparoscopy.

A significant challenge in developing SSR models for TURBT is the limited availability of large video libraries. Unlike laparoscopic procedures, which have benefitted from large public video libraries such as Cholec80 (Twinanda et al., 2017), surgical video libraries for TURBT must be curated *de novo*. The manual annotation of surgical videos is a labor-intensive process, impeding the development of video datasets large enough to achieve high levels of accuracy from SSR models (Hashimoto et al., 2019). Moreover, many surgical procedures are not performed frequently enough to collect sufficient surgical videos to train robust AI algorithms.

Recent advancements in AI technology provide hope that transfer-learning may offer the ability to reduce dataset size requirements for model-training (Neimark et al., 2021a). Similar to a surgeon-in-training’s ability to transfer knowledge and skills learned from one surgical procedure to another, machine learning models can be pre-trained on one surgical procedure and then leverage that pre-training to more efficiently learn a different procedure, thereby reducing dataset requirements. While transfer-learning models pre-trained on one laparoscopic procedure have proven successful in attaining improved SSR accuracy in classification of a different laparoscopic procedure (Eckhoff et al., 2023), it remains unknown whether pre-training on laparoscopic procedures can effectively reduce the dataset requirements for procedures that significantly differ in both temporal and visual features, such as endourologic surgeries.

In order to bridge the progress made in laparoscopic SSR to endoscopic surgery, this study aims to develop a novel computer vision algorithm for automated detection of key steps in TURBT. Through leveraging pre-training on several different laparoscopic procedure types, this study also investigates the feasibility of applying transfer-learning to SSR between procedures that differ greatly in surgical content.

## 2 Methods

### 2.1 Video datasets

A retrospective review was performed to identify patients undergoing TURBT for clinically significant bladder tumors at two tertiary referral centers from December 2021 through December 2022. Videos were included in the dataset if they consisted of TURBT conducted with monopolar or bipolar electrocautery. TURBTs utilizing laser technology as the primary resection modality or en bloc laser tumor resection were excluded, as both were infrequently performed in our dataset.

Surgical video was recorded and stored on a secure cloud-based server using an artificial intelligence surgical video platform (Theator, Inc.). To protect patient confidentiality, an algorithm automatically blurred the surgical video footage when it was outside of the body (Zohar et al., 2020). This study was approved by the Mayo Clinic IRB. All surgical videos were fully de-identified, and the requirement to obtain informed consent was waived by the IRB. The authors did not have access to information that could identify study participants at any point in the study.

All videos in this study were preprocessed in the same manner (Bar et al., 2020). Initially, videos were processed using FFmpeg 3.4.6 on Ubuntu 18.04, and all video streams were encoded with libx264, using 25 frames per second (FPS). The video width was scaled to 480 and the height was determined to maintain the aspect ratio of the original input video. The audio signal was removed from all videos. Segments at the beginning and end of the video not relevant to the procedure were trimmed. Videos were manually annotated by medical image annotators who specialize specifically in surgical video annotation. All annotations were performed with clearly defined and pre-specified criteria for surgical steps. A fellowship-trained urologic oncologist oversaw the annotation process. Every video was annotated by a human annotator, and then independently validated by a second human annotator. In prior studies using this annotation workflow, we have demonstrated a mean inter-rater reliability of 95.82 (standard deviation 3.85) (Khanna et al., 2024). Each second of video footage was annotated with one and only one surgical step.

### 2.2 Definitions of TURBT steps

**Figure 1:**
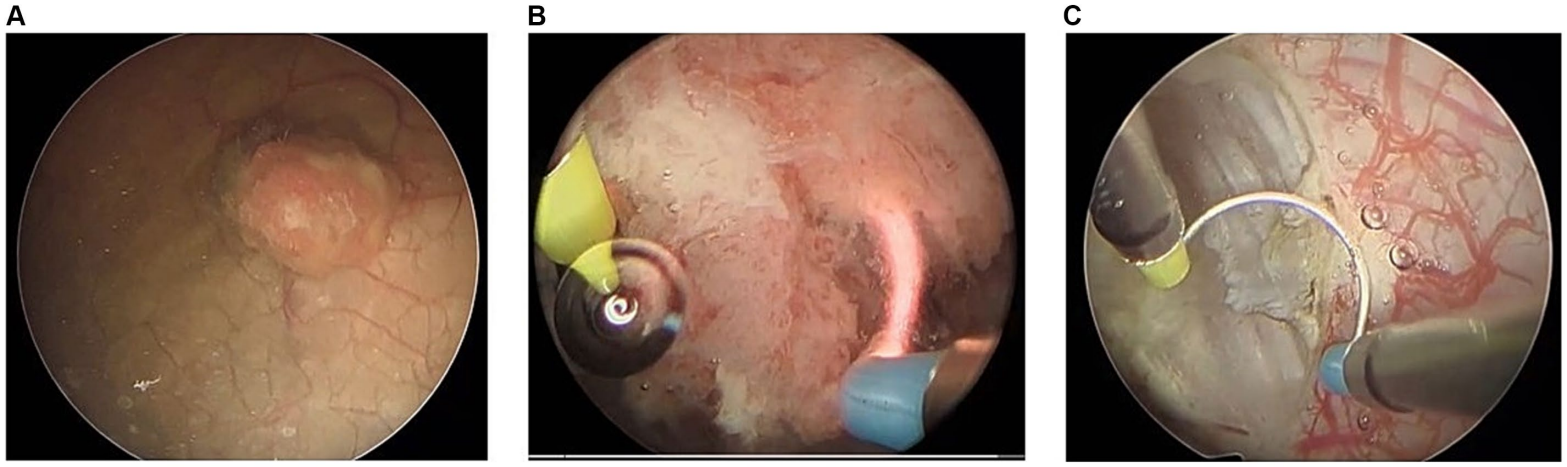
Surgical video footage demonstrating key visual cues associated with each distinct step of surgery. **(A)** Primary evaluation including visual evaluation of tumor, **(B)** resection of bladder tumor, and **(C)** surface coagulation and hemostasis.

Key surgical steps of TURBT, as outlined in Table 1, were defined using expert consensus among fellowship-trained urologic oncologists. These defined steps align with those documented previously in the literature and commonly referenced surgical atlases (Wiesner et al., 2010; Smith et al., 2016). Figure 1 highlights the key visuo-spatial cues and anatomic relationships associated with each distinct surgical step in TURBT.

**Table 1:** Overview of the step definitions of the intravesical portion of TURBT.

TURBT step	Description	Start point	End point
Primary evaluation	Primary endoscopic evaluation, wherein key anatomic landmarks including the location of the bladder trigone, ureteral orifices, visualization of all bladder walls and initial tumor evaluation and identification is performed.	This step is initiated when the endoscope first enters the urethra.	This step ends once resection of a bladder tumor is initiated.
Resection of bladder tumor	Resection of visible bladder tumors. Smaller papillary tumors can often be resected in one swipe at their base, whereas larger sessile tumors can require several swipes.	This step is initiated when bladder tumor resection starts with electrocautery.	This step ends when resection action using electrocautery is definitively stopped and no additional tissue is being resected.
Surface coagulation and hemostasis	The resection site is evaluated for hemostasis. Cauterization of the edges and the base is performed as needed. Bladder emptying should be performed, and the site inspected with flow turned off.	This step is initiated when the resection site is being observed for hemostasis and electrocautery is being used to achieve hemostasis.	This step ends with the exit of the resectoscope from the bladder, which marks the completion of the surgery.

### 2.3 Measures

In determining the accuracy of the AI model, its label determination was compared to the manual human labels. Accuracy was defined as the ratio between the number of seconds of correct prediction to the overall number of seconds in the full-length video.

### 2.4 Step recognition models

The dataset was split into separate sets for training, internal validation, and testing. The algorithm development process involved using the training dataset and periodically evaluating the model’s accuracy on the internal validation dataset. The model was never trained nor exposed to any videos from the test dataset, thus preserving the integrity of the test cohort in assessing model accuracy.

Our TURBT step detection AI tool follows a similar structure to a previously developed algorithm in our group for recognizing surgical steps (Bar et al., 2020). However, the current algorithm is unique to the TURBT cohort. Algorithm development consisted of two overarching elements. Firstly, a deep feature extraction model generated a representation for each second of the surgical video. Second, a temporal model learned to predict surgical steps based on the sequence of learned features from the extraction model. To enhance the performance of our algorithm and to reduce size requirements of our datasets, we utilized a transfer-learning technique from our previous work on laparoscopic cholecystectomy, appendectomy, and sleeve gastrectomy.

The initial stage in algorithm development entailed constructing a feature extraction model. Within this step, we utilized a Video Transformer Network (VTN) to process the complete video as a sequential arrangement of images (frames), spanning from the initial frame to the final frame (Neimark et al., 2021b). The Video Transformer Network (VTN) incorporates attention-based modules to effectively capture spatial and temporal information within the input video. The model underwent fine-tuning specifically for the step-recognition task, with further training carried out utilizing the TURBT video dataset. Once the fine-tuning process was completed, the resulting model was employed as a feature extractor for the TURBT videos. The identified features were subsequently utilized as input for the temporal model.

The temporal model was a Long Short-Term Memory (LSTM) network (Goodfellow et al., 2016). This particular variant of Recurrent Neural Network (RNN) possesses the capability to effectively handle extensive sequences by incorporating the present temporal representation alongside the retention of pertinent historical information, which significantly influences the ultimate predictions of the model. Considering that video data is subjected to post-surgical processing, we employed a bidirectional Long Short-Term Memory (LSTM) architecture to handle the video in dual directions, encompassing both start-to-end and end-to-start processing. The hidden dimension was configured as 128, accompanied by a Dropout layer with a probability of 0.5. A linear layer was then employed to map from the hidden LSTM space to the three TURBT steps. For training, we employed a cross-entropy loss function and trained the network for 100 epochs, utilizing an SGD optimizer with a learning rate of 10^−2^.

## 3 Results

A total of 300 full-length TURBT videos were included, which were subdivided into training (*n* = 179), internal validation (*n* = 44), and test (*n* = 77) cohorts. Each surgical video contained all three intravesical steps of TURBT. The mean duration of surgical videos in the dataset was 32.21 min with a SD of 25.68 min. Table 2 details the length of each individual TURBT step.

**Table 2:** Median duration of each step of the TURBT among the train-test splits of the video dataset.

	Number of videos	Median operative duration (minutes ± IQR)	Median duration primary evaluation step (minutes ± IQR)	Median duration bladder tumor resection step (minutes ± IQR)	Median duration surface coagulation step (minutes ± IQR)
Full dataset	300	23.96 (14.13–41.31)	3.75 (1.8–7.22)	11.09 (5.25–24.47)	6.33 (3.85–11.03)
Train	179	24.18 (14.23–43.46)	3.9 (1.78–8.0)	11.45 (5.42–21.85)	6.82 (3.87–11.61)
Validation	44	23.1 (15.08–33.39)	3.57 (2.02–5.62)	15.08 (7.21–22.58)	5.35 (3.41–8.83)
Test	77	25.38 (14.03–48.5)	3.47 (1.76–7.28)	9.57 (4.74–27.15)	6.18 (4.04–10.1)

**Figure 2:**
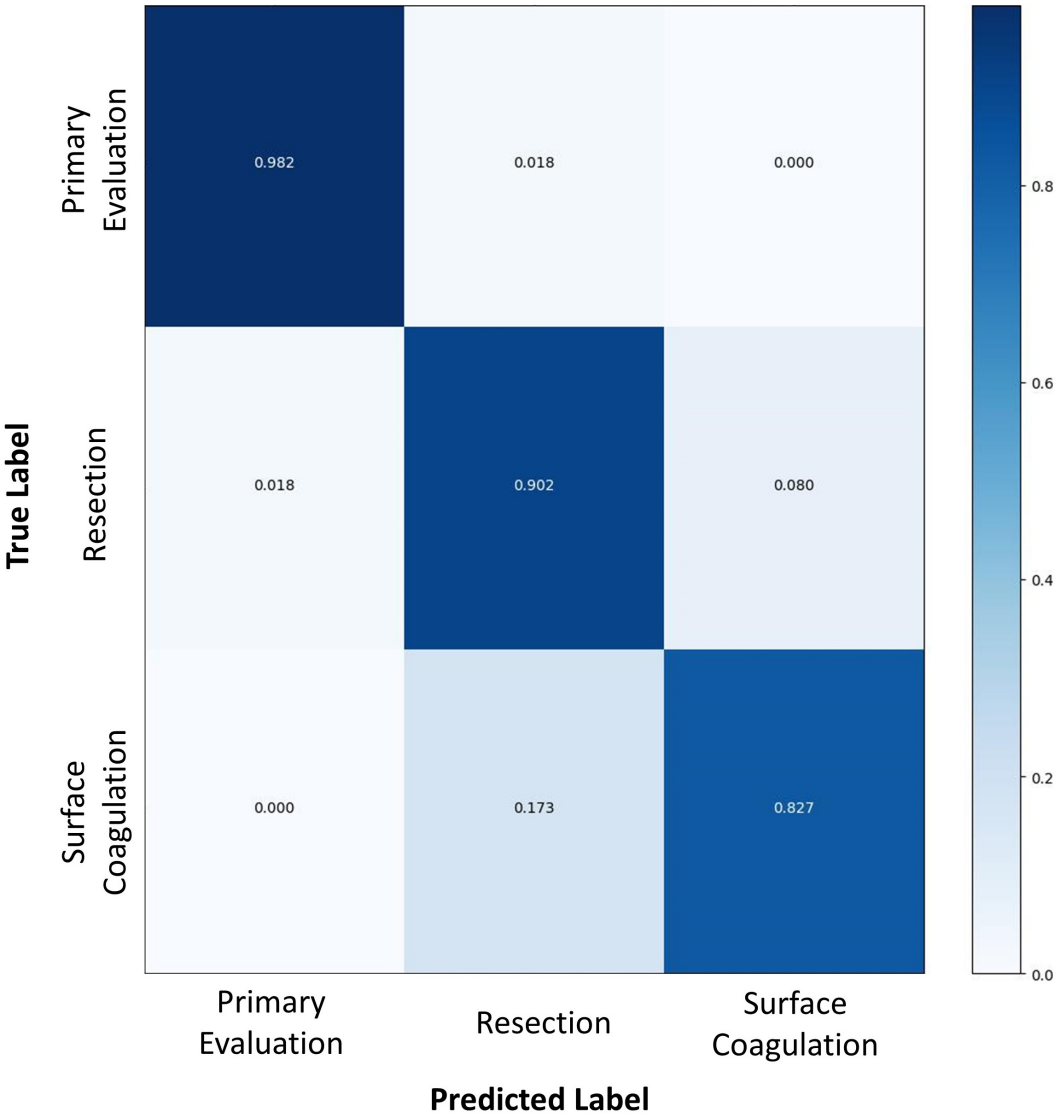
A confusion matrix comparing the surgical step predicted by the AI algorithm (horizontal axis) to the true labels as determined by human video annotation (vertical axis). Numbers in dark blue represent the accuracy of AI for predicting that particular surgical step, whereas numbers in white or light blue represent the proportion of inaccurate predictions for each surgical step.

Overall accuracy for the complete AI model in determining TURBT step on the test dataset was 89.6%. Per-step accuracy for (1) primary evaluation (2), tumor resection, and (3) hemostasis was 98.2, 90.2, and 82.7%, respectively. As demonstrated in Figure 2, errors in labeling were most often attributed to a misclassification between temporally adjacent steps.

## 4 Discussion

We developed an AI-powered computer vision algorithm for automated detection of key surgical steps during TURBT with high accuracy. To our knowledge, this represents the first demonstration of a comprehensive surgical step recognition algorithm in the field of endourology. Through leveraging pre-training on laparoscopic procedures toward SSR of endoscopic surgery, the algorithm presents a novel application of transfer-learning to entirely different surgical modalities characterized by substantial variation in visual and temporal content from the pre-training data. The overall accuracy of this model is concordant with those previously reported for laparoscopic procedures, thus providing evidence for the versatility and applicability of SSR beyond its initial application in laparoscopic surgery to utility in surgical endoscopy (Cheng et al., 2022; Jumah et al., 2022; Takeuchi et al., 2022, 2023).

This study highlights the ability of pre-trained SSR models to extrapolate overarching patterns across diverse procedures, thereby reducing the need for extensive training datasets and improving the efficiency of model development. During an early iteration of the current model developed from a preliminary cohort of only 108 full-length TURBT videos (with a train-test-validate split of 62, 19, and 27 respectively), the model’s overall accuracy was 86.3%, similar to the 89.6% overall accuracy of the final model developed from the complete 300 video dataset. This underscores the significant potential of applying transfer learning techniques in training new SSR models, including across entirely different surgical modalities. Endourologic and laparoscopic procedures differ greatly in both the medium of operation, anatomical targets, instruments used, actions performed, and the order in which maneuvers are performed. Despite vast differences in data characteristics, the current SSR model effectively utilized shared temporal and visual patterns between laparoscopic and endourologic surgical steps to achieve high accuracy.

Application of transfer-learning may reduce the need for curation of large datasets, which must be laboriously annotated by surgical experts. Training on a wide array of surgical procedures that exhibit disparate data characteristics may allow for the extension of SSR to procedures that are less commonly performed and accordingly lack large-scale video data. Moreover, in the future, context-aware computer-assisted surgery (CA-CAS) systems are predicted to aid surgeons in real-time intraoperative decision-making (Bodenstedt et al., 2020). Thus, it is critical for CA-CAS systems to leverage prior training to successfully interpret individual surgeon techniques, unexpected surgical events, and unique patient anatomy. Not only is transfer-learning a promising strategy to increase the robustness of SSR models, but it also serves as a conceptual framework for further development of future CA-CAS systems.

The accuracy of this spatiotemporal model fared well in the first two steps of the procedure, achieving 98.2 and 90.2% accuracy, respectively. However, it exhibited lower accuracy on the last step of surface coagulation and hemostasis (82.7%). This step was a considerably short step with a median time of 6.3 min. Previous research has shown that SSR classification errors occur most frequently due to misclassification of temporally adjacent steps, particularly at the beginning or end of a step (Bar et al., 2020). Given the relatively short length of the surface coagulation and hemostasis step, the transitional boundary between this step and the preceding step accounts for a relatively greater proportion of the final step’s overall duration. Accordingly, errors in step classification attributable to temporal shift likely contributed to an inflated SSR inaccuracy for this model. Moreover, this step encompasses substantial content variance depending on the extent of bleeding, ranging from simple observation of the surgical resection bed to the requirement for extensive hemostasis involving electrocautery. The use of electrocautery for hemostasis is visually similar to the use of electrocautery for resection of small residual tumors in the prior step, thus the boundary between the two steps is further obfuscated by similarities in visual features of tasks performed.

The conditions under which transfer-learning is appropriate and feasible remain to be established. Eckhoff and Ban et al. found that transfer-learning demonstrates limitations in recognizing steps with low procedural overlap and resemblance of visual features to the pre-training dataset (Eckhoff et al., 2023). This is likely attributable to a combination of low feature similarities to the pre-training dataset and due to relative underrepresentation of the step in the training dataset.

Indeed, laparoscopic procedures exhibit notable disparities in both spatial and temporal patterns when compared to endoscopic procedures. Unique characteristics define the beginning and ending points in laparoscopic procedures, exemplified by the clear and obvious distinctions between temporally adjacent steps like urethral transection and vesicourethral anastomosis in radical prostatectomy. These two steps differ in terms of the instruments employed (scissors versus suturing needles), camera angles, and anatomical relationships (Khanna et al., 2023). In contrast, the features observed between resection and coagulation steps of TURBT display close similarities, as they involve the same tools, similar actions, and the same anatomical region of the bladder. This distinction highlights the difficulty in training SSR models for endourologic procedures, thus providing context to the relatively high accuracy of the current model. Future iterations of this model could strive to improve detection of the surface coagulation step by attempting to train the model to distinguish between resectoscope activation for tumor removal (which involves a dynamic surgical instrument and active resection of tissue) versus for tissue coagulation (which often involves a more static surgical instrument and no visible tissue being removed from the bladder wall).

The applications of SSR models are broad and numerous. Modern laparoscopic, endoscopic and robotic surgery produces vast amounts of video footage. However, much of this videographic information is lost. Analysis of surgical videos currently requires resource-intensive review by surgeons. Through automatic video annotation, SSR can greatly expedite the review process, enabling surgeons to quickly assess crucial aspects of a procedure. This technology has practical applications in facilitating surgical documentation (Khanna et al., 2023), as an integral tool for surgical training (Garrow et al., 2021), and to augment operating room logistics and staffing (Garrow et al., 2021).

It has been shown that intraoperative performance is strongly linked to post-operative outcomes (Birkmeyer et al., 2013). Based on an intuitive understanding of this principle, surgical research has often operationalized surgical performance by relying on largely surrogate metrics, such as length of surgery, estimated blood loss, or hospital length of stay. While these metrics have been associated with post-operative outcomes in numerous studies, ultimately they are still only surrogates for what actually transpires in the operating room. In comparison, video-based metrics provide more granular insight into intraoperative workflow, and may be more strongly linked to postoperative outcomes. As highlighted by a series of recent studies by Kiyasseh et al., further insight into intraoperative details can ultimately assist surgeons in refining their skillsets and improving surgical outcomes (Kiyasseh et al., 2023a,b). The current study of SSR in TURBT builds the foundation for future efforts to glean insights into endourology surgical practice and the impact of intraoperative events on postoperative outcomes.

Strengths of this study include the use of data from two tertiary medical centers. Training a SSR model with a limited dataset could lead to over-fitting and subsequently reduce the generalizability of the model. Therefore, videos from different medical institutions and surgeons should be included to ensure adequate heterogeneity in the dataset. Furthermore, this study introduces a computer-vision-based algorithm that is trained exclusively on visual data. While previous research has made extraordinarily promising progress in the application of deep learning techniques to interpret intraoperative content (Hung et al., 2019; Ma et al., 2022), many prior studies rely on kinematic data collected through additional hardware that tracks surgical tool trajectories based on angles of instrument joints, economy of motion, and instrument speed (Hung et al., 2018). In contrast, by relying solely on visual data, this SSR model offers a practical advantage in terms of implementation. It eliminates the need for significant capital investment in hardware acquisition and can be seamlessly applied to any surgical platform, thereby reducing the barrier to adoption and facilitating its integration into existing operating room settings. Prior studies utilizing data inputs from the da Vinci robotic surgical platform (Intuitive, Inc.) cannot be applied beyond robotic surgery into other valuable domains, such as laparoscopic or endoscopic surgery.

Study results should be interpreted in the context of methodological limitations. The steps of TURBT were split into three steps during the intravesical part of TURBT, but there is potential to divide TURBT into a different schema of steps. Specifically, there was consideration to split the resection step into distinct steps for active tumor resection and bladder chip collection. However, it was determined that active tumor resection and bladder chip collection represented repetitive tasks within the overarching objective of removing all visible tumors, thus these were deemed to be encompassed into a single surgical step. Additionally, the training dataset for this model excluded laser resection and en bloc resection, which do not represent the standard of care and are performed infrequently. Nonetheless, it is important to acknowledge that the practices, techniques and surgical videos used in this study may have limited application to surgeons who utilize those techniques. However, video data for this study incorporated several surgeons with a variety of different surgical techniques, so it is anticipated that the external validity of this model will be adequate. Further, we demonstrated that utilizing transfer learning from laparoscopy to endoscopy resulted in a high-accuracy model for TURBT, but we did not develop a separate TURBT model without transfer learning to serve as a comparison. Future efforts should include a comparison that does not employ transfer learning, as this would help further our understanding of the incremental benefits attributable to transfer learning approaches. Finally, the current dataset had very few examples of rare surgical events, such as bladder perforation or resection of the ureteral orifices, which provides an opportunity for further refinement of this algorithm in larger datasets in the future.

## 5 Conclusion

In conclusion, this study presents a novel AI surgical step recognition tool capable of automatically classifying the steps of a TURBT based solely on surgical video. This technology leveraged transfer-learning by pre-training on laparoscopic procedures to reduce the size of TURBT datasets required for the current study. This technology has numerous potential applications in surgical education, operating room logistics and operations, and correlating intraoperative events with surgical outcomes, all of which warrant further study.

## Data availability statement

The datasets presented in this article are not readily available because the data that support the findings of this study cannot be shared publicly due to concerns surrounding the privacy of individuals that participated in the study. Data access requests can be submitted through the corresponding author to Mayo Clinic Rochester and are subject to institutional approval. Requests to access the datasets should be directed to AK, khanna.abhinav@mayo.edu.

## Ethics statement

The studies involving humans were approved by Mayo Clinic Institutional Review Board. The studies were conducted in accordance with the local legislation and institutional requirements. The ethics committee/institutional review board waived the requirement of written informed consent for participation from the participants or the participants’ legal guardians/next of kin because the study did not adversely affect the rights and welfare of participants.

## Author contributions

ED: Data curation, Formal analysis, Funding acquisition, Investigation, Resources, Visualization, Writing – original draft, Writing – review & editing, Methodology, Validation. MT: Conceptualization, Data curation, Investigation, Methodology, Project administration, Resources, Supervision, Writing – original draft, Writing – review & editing. AA: Data curation, Investigation, Methodology, Visualization, Writing – original draft, Writing – review & editing. MZ: Data curation, Formal analysis, Methodology, Validation, Visualization, Writing – original draft, Writing – review & editing. OB: Data curation, Formal analysis, Investigation, Supervision, Visualization, Writing – original draft, Writing – review & editing. DB-A: Data curation, Formal analysis, Investigation, Methodology, Software, Validation, Visualization, Writing – original draft, Writing – review & editing. LM: Investigation, Methodology, Project administration, Resources, Supervision, Writing – original draft, Writing – review & editing. DL: Investigation, Methodology, Project administration, Resources, Visualization, Writing – original draft, Writing – review & editing. RA: Investigation, Methodology, Project administration, Resources, Supervision, Writing – original draft, Writing – review & editing. AM: Investigation, Methodology, Project administration, Resources, Supervision, Writing – original draft, Writing – review & editing. DE: Investigation, Methodology, Project administration, Resources, Supervision, Writing – original draft, Writing – review & editing. SB: Investigation, Methodology, Project administration, Resources, Supervision, Writing – original draft, Writing – review & editing. TW: Data curation, Formal analysis, Investigation, Methodology, Project administration, Resources, Software, Supervision, Validation, Visualization, Writing – original draft, Writing – review & editing. DA: Data curation, Formal analysis, Investigation, Methodology, Project administration, Resources, Software, Supervision, Validation, Visualization, Writing – original draft, Writing – review & editing. AK: Project administration, Resources, Supervision, Visualization, Writing – original draft, Writing – review & editing, Conceptualization, Data curation, Funding acquisition, Investigation, Methodology.
